# Do NERICA rice cultivars express resistance to *Striga hermonthica* (Del.) Benth. and *Striga asiatica* (L.) Kuntze under field conditions?

**DOI:** 10.1016/j.fcr.2014.10.010

**Published:** 2015-01

**Authors:** Jonne Rodenburg, Mamadou Cissoko, Juma Kayeke, Ibnou Dieng, Zeyaur R. Khan, Charles A.O. Midega, Enos A. Onyuka, Julie D. Scholes

**Affiliations:** a Africa Rice Center (AfricaRice), East and Southern Africa, P.O. Box 33581, Dar es Salaam, Tanzania; b Mikocheni Agricultural Research Institute (MARI), Dar es Salaam, Tanzania; c Africa Rice Center (AfricaRice), 01 BP2031, Cotonou, Benin; d International Centre of Insect Physiology and Ecology (ICIPE), P.O. Box 30772, Nairobi 00100, Kenya; e International Crops Research Institute of the Semi-Arid Tropics (ICRISAT), Eastern and Southern Africa, P.O. Box 39063, Nairobi, Kenya; f Department of Animal and Plant Sciences, University of Sheffield, Sheffield S10 2TN, UK

**Keywords:** Parasitic weeds, Tolerance, Upland rice, *Oryza sativa*, *Oryza glaberrima*, Africa

## Abstract

The parasitic weeds *Striga asiatica* and *Striga hermonthica* cause high yield losses in rain-fed upland rice in Africa. Two resistance classes (pre- and post-attachment) and several resistant genotypes have been identified among NERICA (New Rice for Africa) cultivars under laboratory conditions (in vitro) previously. However, little is known about expression of this resistance under field conditions. Here we investigated (1) whether resistance exhibited under controlled conditions would express under representative *Striga*-infested field conditions, and (2) whether NERICA cultivars would achieve relatively good grain yields under *Striga*-infested conditions. Twenty-five rice cultivars, including all 18 upland NERICA cultivars, were screened in *S. asiatica*-infested (in Tanzania) and *S. hermonthica*-infested (in Kenya) fields during two seasons. Additionally, a selection of cultivars was tested in vitro, in mini-rhizotron systems. For the first time, resistance observed under controlled conditions was confirmed in the field for NERICA-2, -5, -10 and -17 (against *S. asiatica*) and NERICA-1 to -5, -10, -12, -13 and -17 (against *S. hermonthica*). Despite high *Striga*-infestation levels, yields of around 1.8 t ha^−1^ were obtained with NERICA-1, -9 and -10 (in the *S. asiatica*-infested field) and around 1.4 t ha^−1^ with NERICA-3, -4, -8, -12 and -13 (in the *S. hermonthica*-infested field). In addition, potential levels of tolerance were identified in vitro, in NERICA-1, -17 and -9 (*S. asiatica*) and in NERICA-1, -17 and -10 (*S. hermonthica*). These findings are highly relevant to rice agronomists and breeders and molecular geneticists working on *Striga* resistance. In addition, cultivars combining broad-spectrum resistance with good grain yields in *Striga*-infested fields can be recommended to rice farmers in *Striga*-prone areas.

## Highlights

• *Striga* spp. resistance in NERICA cultivars, observed in vitro, was confirmed in situ.
• NERICA-2, -10 (*S. asiatica*) and -10, -17 (*S. hermonthica*) are most resistant.
• NERICA-1, -17, -9 (*S. asiatica*) and -1, -17, -10 (*S. hermonthica*) show tolerance.
• Some NERICA cultivars yielded 1.5–2 t ha^−1^ despite *Striga* spp. infestation.

## 1 Introduction

In sub-Saharan Africa (SSA), rice is an increasingly important cereal crop (Seck et al., 2012) in rain-fed agro-ecosystems. Of the total area under rice in SSA, 32% can be characterized as rain-fed upland, with average estimated yields of around 1.2 t ha^−1^ (Diagne et al., 2013). The extremely low productivity in these rain-fed upland environments is caused by a myriad of bio-physical and socio-economic constraints (e.g. Balasubramanian et al., 2007). Major production constraints for smallholder farms in rain-fed agro-ecosystems in Africa are drought, poor soil fertility and weeds (Waddington et al., 2010). Weed species that are frequently observed on these poorly fertile and drought-prone soils are those of the parasitic Orobanchaceae family (e.g. Parker, 2009, Rodenburg and Johnson, 2009). *Striga* spp., in particular *Striga hermonthica* (Del.) Benth. and *Striga asiatica* (L.) Kuntze, are the most wide-spread and economically important species of parasitic weeds in cereal cropping systems (e.g. Mohamed et al., 2001, Rodenburg et al., 2010, Spallek et al., 2013).

*Striga* species negatively affect the growth and yield of the crops they infect (e.g. Webb and Smith, 1996, Frost et al., 1997, Gurney et al., 1999). However, the extent of these negative effects is a function of the environment and the genetic make-up of the host and parasite. Host plant genotypes may show various levels, mechanisms and combinations of resistance and tolerance to *Striga* species, where resistance (antonym: susceptibility) reduces the *Striga* infection levels and tolerance (antonym: sensitivity) alleviates the effects of infection (e.g. Yoder and Scholes, 2010, Rodenburg and Bastiaans, 2011). Some rice cultivars (e.g. *Oryza sativa* cultivars IR47255-B-B-5-4, IR49255-B-B-5-2, Nipponbare and IR64 and *O. glaberrima* cultivars ACC102196, Makassa, CG14 and IG10) exhibit good resistance against some ecotypes of *S. hermonthica* whilst other cultivars (e.g. IAC165 and Koshihikari) are susceptible (Harahap et al., 1993, Johnson et al., 1997, Gurney et al., 2006, Kaewchumnong and Price, 2008, Swarbrick et al., 2009). High genetic variability has also been observed among different species and ecotypes (i.e. genetically distinct populations within a species) of the parasite (Botanga et al., 2002, Huang et al., 2012), making *Striga* management complex as the resistance found in some cultivars may be overcome by a small subset of *Striga* individuals within the seed bank leading to the development of a virulent population over time (e.g. Rodenburg and Bastiaans, 2011, Kountche et al., 2013).

Nevertheless, the use of *Striga* resistant cultivars is widely considered as one of the most suitable and effective control options for resource-poor farmers (Haussmann et al., 2000). However, very few rice cultivars are known that combine resistance to *Striga* species and/or ecotypes, with adaptability to African upland rice growing environments (Rodenburg et al., 2010). This gap can potentially be filled by a group of inter-specific, NERICA (‘New Rice for Africa’) rice cultivars which are widely distributed and adopted across Africa (Diagne, 2006, Kijima et al., 2006, Wopereis et al., 2008). The NERICA cultivars are the progeny of crosses between the African rice species *Oryza glaberrima* (Steud.) and the Asian rice species *Oryza sativa* (L.). They were generated to combine the weed competitiveness and resilience to abiotic and biotic stresses of the African rice species with the high yield and grain quality of the Asian rice species (Jones et al., 1997a, Jones et al., 1997b).

Recently, Jamil et al. (2011a) and Cissoko et al. (2011) evaluated the 18 upland NERICA cultivars released so far (and their parental genotypes) for pre- and post-attachment resistance respectively, against different *Striga* species and ecotypes under controlled environment conditions. Pre-attachment resistance entails all mechanisms that hamper the development of the parasite before attachment to the host root. Post-attachment resistance are all mechanisms that prevent or hamper the attached parasite to establish the necessary xylem–xylem connection with the host root. Some of the NERICA cultivars displayed an excellent degree of post-attachment resistance against ecotypes of *S. hermonthica* and *S. asiatica* (Cissoko et al., 2011). Also, variation in the quantity and type of strigolactone production, and consequently the ability to germinate *S. hermonthica* seeds was found among this group of rice cultivars. Those that produced low amounts of strigolactones showed good levels of pre-attachment resistance (Jamil et al., 2011a, Jamil et al., 2011b). Some cultivars, e.g. NERICA-1, -3, -4, -12 and -17, showed excellent combinations of pre- and post-attachment resistance which may increase the durability of resistance under field conditions. However, whilst we now know which of the NERICA cultivars show resistance to *Striga* spp. and ecotypes under highly controlled growth conditions, we know much less about the impact of environment on the expression of resistance, hence whether the resistance exhibited by some cultivars in vitro (laboratory) will be effective in situ (field). In addition, apart from a study by Atera et al. (2012) where a selection of four NERICA cultivars was grown under *S. hermonthica*-infested conditions, there is no published information about the adaptability and yield performance of different NERICA cultivars under *Striga*-infested field conditions.

The objectives of this study were therefore to determine (1) whether the resistance of the NERICA cultivars identified under controlled environment conditions (i.e. pre- and/or post-attachment resistance) is exhibited as reduced above-ground parasite numbers in *S. hermonthica* and *S. asiatica*-infested fields in Africa, and (2) whether cultivars that exhibit good resistance also have good rice grain yields. To achieve this, two seasons of field screening trials were conducted with all 18 upland NERICA cultivars, their parents, and known susceptible, resistant and local check cultivars, in two different locations, at Kyela, Tanzania (where fields are infested with *S. asiatica*) and at Mbita, Kenya (under *S. hermonthica* infestation). In addition, the resistance of selected cultivars was investigated under controlled environment conditions following infection with the *Striga* ecotypes obtained from the field sites.

## 2 Materials and methods

### 2.1 Plant materials

All 18 interspecific upland NERICA rice cultivars, NERICA-1 to -18, their *O. glaberrima* parent, CG14, and *O. sativa* ssp. japonica parents, WAB56-104, WAB56-50 and WAB181-18, were grown in *Striga*-infested plots at Kyela, Tanzania under *S. asiatica* infestation, and at Mbita, Kenya under *S. hermonthica* infestation. In addition to these 22 cultivars, in Kyela, two traditional and locally popular cultivars, Supa India (synonym: Kilombero; included as locally adapted but *Striga*-susceptible check) and Mwangulu (included as locally adapted and *Striga*-resistant check), and an international cultivar originally from Brazil, IAC165 (*Oryza sativa* ssp. Japonica; included as *Striga*-susceptible check), were selected, making a total of 25 cultivars. For the trials in Mbita, Mwangulu was replaced by the cultivar IR49255-B-B-5-2 (*O. sativa* ssp. Indica; included as resistant check) (identified by Harahap et al., 1993, Johnson et al., 1997). Seeds of all rice cultivars were obtained from the Africa Rice Center (AfricaRice), Cotonou, Benin except for Supa India and Mwangulu, which were supplied by the Agricultural District Office of Kyela. Seeds of *S. asiatica* and *S. hermonthica* were collected in the previous season from plants parasitizing rice at Kyela, Tanzania (Sa-Kyela) and maize at Mbita, Kenya (Sh-Mbita) in farmer’ fields surrounding the experimental field sites. These seeds were used to supplement the soil seed bank in the field trials as well as for the controlled environment studies.

### 2.2 Experimental sites

The *S. asiatica* field screening trials were conducted during the rainy seasons (February–July) of 2011 and 2012 in Mbako (9°35′ S–33°48′ E; 525 m a.s.l.), a village approximately 15 km from Kyela, in Kyela district, Mbeya region in southern Tanzania (Table 1). The district is part of the Southern Highlands and located in the west arm of the African Rift Valley on the shores of Lake Malawi. Kyela district is a *S. asiatica*-infested upland rice-growing area. Since no experimental station in Africa exists where screening work in *S. asiatica*-infested fields can be conducted, we opted to execute this work in two already infested farmers’ fields in this *S. asiatica* endemic area. Cumulative rainfall measured in the field during the trials was 2474 mm in 2011 and 2499 mm in 2012 (Table 1). In 2012 two field trials were conducted. The first was carried out in the same field as the 2011 trial and is referred to as Kyela 2012-1. This field trial was duplicated in a second field in the same village, approximately 1 km from the first field and is referred to as Kyela 2012-2.

**Table 1:** Overview of experimental conditions of the field trials conducted at Kyela, Tanzania (2011 and 2012), and at Mbita, Kenya (2010 and 2011).

Location	Kyela–Tanzania (*S. asiatica*)			Mbita–Kenya (*S. hermonthica*)	
	(9°37′30′′ S–33°52′30′′E)			(0°42′82′′ S–34°20′53′′ E)	
Altitude (m a.s.l.)	525			1141	
Year	2011	2012-1	2012-2	2010	2011
Season/Period	Single rain/Feb–Jun			Short rain/Sep–Jan	Long rain/Mar–Aug
Cumulative rainfall (mm)	2474	2499		281^a^	615
Sowing dates	09/02/211	22/02/2012	29/02/2012	17/09/2010	17/03/2011
Cultivars	24 + Mwangulu			24 + IR49255-B-B-5-2	
Net plot size (m^2^)	117.25 m^2^			86 m^2^	
Net sub-plot size (m^2^)	4.69 m^2^			3.44 m^2^	
Fertilizer application	100 kg ha^−1^ N-P-K: 20-10-10			50 kg ha^−1^ N-P-K: 17-17-17	
*Striga* infestation density (m^−2^)	0.91 g (243,000 seeds^b^)			0.60 g (85,000 seeds)	
Soil parameters					
Sand:silt:clay	63:14:23	63:14:23	63:13:25	–	–
pH	5.21	4.80	4.75	5.70	5.95
N (%)	0.10	0.11	0.11	0.60	1.46
P (ppm)	4.11	6.8	5.9	–	–
K (ppm)	230	218	229	–	–

a Supplementary irrigation was provided.

b Seed weights according to Parker and Riches (1993).

The *S. hermonthica* field screening trials were conducted during the short rainy season of 2010 (September to January) and the long rainy season of 2011 (March to August) at the farm of the International Center of Insect Physiology and Ecology (ICIPE) at Mbita (0°42′ S–34°20′ E; around 1141 m a.s.l.), located on a peninsula in Lake Victoria, Suba District, western Kenya (Table 1). The trial was laid out on a heavily *Striga*-infested field at the west-side of the peninsula that was formerly under sorghum – cassava rotation. Cumulative rainfall was 281 mm in 2010 (short rain) and 615 mm in 2011 (long rain) (Table 1). Rainfall data were obtained from ICIPE's meteorological station 500 m from the field. Supplementary irrigation (by sprinkler) was applied when rainfall was insufficient (in Mbita only).

### 2.3 Experimental design, plot sizes and field preparation

All field trials were laid out in a 5 × 5 lattice design with six replicates. At Kyela each plot, representing an individual cultivar, measured 1.25 m × 3.75 m (4.69 m^2^) and contained 5 rows of 15 hills with a plant distance of 0.25 m × 0.25 m (Table 1). At Mbita each plot measured 1.25 m × 2.75 m (3.44 m^2^) with 5 rows of 11 rice planting hills with the same plant distance as in Kyela (Table 1). Plots were separated by one open row of 0.25 m to avoid neighbor effects and to allow easy access. Each replicate was separated by a 1.25 m alley.

Each plot received supplementary *Striga* seeds mixed with white sand. An amount of 4.25 g of *S. asiatica* seed (germination rate: 55–65%) mixed with 450 g sand at Kyela and 2.07 g of *S. hermonthica* seed (germination rate: 75–80%) in 450 g sand at Mbita were used, resulting in an infestation density of 0.9 g seed m^−2^ at Kyela (approx. 146,000 viable *S. asiatica* seeds) and 0.6 g seeds m^−2^ at Mbita (approx. 66,000 viable *S. hermonthica* seeds). The mixture was broadcast and incorporated into the upper 5–10 cm of soil using short-handled-hoes, prior to rice sowing. Implications of additional *Striga* infestation in the selected farmers’ fields in Kyela were carefully explained to the farmers owning the land, during discussions prior to the experimental seasons. Measures to restore the original conditions were presented and our technical and financial assistance to achieve this was guaranteed.

In all trials, rice was directly sown at approximately 6 seeds per hill, and thinned to 2–3 plants per hill 25 days after sowing (DAS). To arrive at the desired plant density, in some cases gap filling was carried out by using supplemental plants from a rice nursery planted at the edge of the field on the same sowing date. From sowing onwards, each trial was regularly hand weeded (at least every 2–3 weeks) to remove all weeds other than *Striga*. At both sites fertilizer was applied at 35 DAS. In Kyela N-P-K (20-10-10) was applied at an equivalent rate of 100 kg ha^−1^, while at Mbita, with relatively nutrient-rich soils, N-P-K (17:17:17) was applied at a rate of 50 kg ha^−1^ (Table 1).

### 2.4 Experimental measurements

The number of above-ground *Striga* plants in each plot, emerged within the central area comprising 27 rice hills, was counted weekly in the Mbita trials, and in Kyela at 57, 85 and 114 DAS (2011), 49, 68, 102 and 118 DAS (2012-1) and 47, 95 and 113 DAS (2012-2). These data enabled the assessment of the maximum number of above-ground *Striga* plants (*NSmax*), which is a reliable measure for *Striga* resistance in the field, following Rodenburg et al. (2005). At harvest emerged *Striga* plants within each observation area of 27 hills in each plot were collected, dried and weighted for the assessment of *Striga* biomass, as an additional resistance measure. At harvest, rice panicles were harvested from the same central 27 hills of each plot. Rice panicles were air-dried for 2 weeks after which rice grains were separated from the panicles and weighed. Grain moisture content was assessed, using a digital grain moisture meter of SATAKE (Model SS-7), to correct rice grain dry weights to 14% moisture.

### 2.5 Phenotyping of *Striga* resistance levels under controlled environment conditions

To determine the impact of the field environment on the resistance ranking of the NERICA cultivars, a subset of the cultivars was phenotyped for post-attachment resistance under controlled environment conditions at the University of Sheffield using the same ecotypes of *S. hermonthica* (Sh-Mbita) and *S. asiatica* (Sh-Kyela) present at the field sites. In addition, the tolerance of these cultivars was assessed as described by Cissoko et al*.* (2011). Six-day-old single rice seedlings were transferred to rhizotrons, which consist of 25 cm × 25 cm × 2 cm perspex containers packed with vermiculite covered by a 100 μm polyester mesh, with openings at the top and bottom to allow shoot growth and drainage. Ten days later the rice plants were infected with 12.5 mg of germinated *S. hermonthica* seeds or 20 mg of germinated *S. asiatica* seeds (Cissoko et al., 2011). Uninfected control plants were treated in a similar manner but without the *Striga* seeds. Four replicates were evaluated for each cultivar × *Striga* sp. combination. The cultivars tested were NERICA-1, -7, -9, -10 and -17, CG14, WAB56-104, WAB56-50, WAB181-18, IAC165 and Supa India. Quantification of post-attachment resistance levels was based on mean parasite dry biomass per host root system for the different cultivars. Host tolerance was assessed by plotting the relative host plant biomass of infected plants (i.e. the biomass of parasite infected plants as percentage of the biomass of parasite-free control plants) against the *Striga* infection level, expressed as the total biomass of the parasitizing plants collected from the host roots.

### 2.6 Statistical analyses

Prior to analyses, data were checked for homoscedasticity and normality following Sokal and Rohlf (1995). Following these tests, field data on rice grain and *Striga* dry weights were analyzed using a Linear Mixed Model. We tested whether there was a significant Trial × Cultivar interaction effect for both locations (Kyela and Mbita). We first performed a log-likelihood ratio test for the homogeneity of variance and when the variance was not constant, we combined the data taking into account the heterogeneity of the variances. When the Trial × Cultivar interaction effect was significant (*P* < 0.05), we fitted a model for each trial separately, where Cultivar was considered as fixed effect and Block, nested into Replicate, and Replicate as random effects. For analyses of maximum above-ground *Striga* numbers (*NSmax*) a Generalized Linear Mixed Model (McCullagh and Nelder, 1989) was used under the assumption of a Poisson distribution. Standard Errors of Differences of Means (SED), LS means and associated standard errors were computed. A Squared Euclidian Distance matrix was computed based on LS means and Ward's clustering procedure (Ward 1963), in which incremental sums of squares as fusion criteria, were applied using a hierarchical agglomerative clustering (Kettenring, 2006). Three measures were used for the validation of the results of the cluster analysis (1) Connectivity (Handl et al., 2005), (2) Dunn Index (Dunn, 1974), and (3) Silhouette Width (Rousseeuw, 1987). Each of these measures evaluates the hierarchical clustering while varying the number of clusters. The optimum number of clusters, provided by at least two of these measures, was presented here. This facilitated clustering of the cultivars in statistically distinct groups, based on *Striga* field resistance and rice grain yield under *Striga*-infested conditions. Spearman rank correlations were calculated between LS means of *NSmax* and *Striga* dry weights and between LS means of *NSmax* and rice yields. The rhizotron data were analyzed following checks for homoscedasticity and normality. ANOVAs were conducted followed by a comparison of means using Tukey's honest significant difference test. All field data were analyzed using the statistical package Genstat (v. 11), the cluster analysis was performed using the clValid package (Brock et al., 2008) of the R software version 3.1.1 (R-Core-Team, 2014) and the rhizotron data were analyzed using Minitab (v. 15).

## 3 Results

### 3.1 How resistant are the NERICA cultivars to *S. hermonthica* and *S. asiatica*?

For both locations (Kyela and Mbita) the Trial × Cultivar interaction effects on maximum above-ground *Striga* numbers were highly significant (*P* < 0.0001), and therefore data were analyzed separately for each field trial. Rice cultivar had a highly significant effect (*P* < 0.001) on the maximum number of emerged *S. asiatica* and *S. hermonthica* (*NSmax*) in all screening trials (Table 2). Rice cultivar also significantly affected *Striga* dry weights at harvest (except in the Kyela 2012-1 trial).

**Table 2:** Variance components analysis (F-stat. and F-prob.) and standard errors of differences of means (SED) of cultivar effects on rice grain dry weights (rice grain DW), maximum above-ground *Striga* numbers (*NSmax*) and above-ground *Striga* biomass (dry weights) (*Striga* DW) at harvest, obtained from *S. asiatica* (Kyela) and *S. hermonthica* (Mbita) infested fields during two seasons per location.

*Striga* sp.^a^	Trial	df	Rice grain DW			*NSmax*^b^			*Striga* DW		
			F-stat.	F-prob.	SED	F-stat.	F-prob.	SED	F-stat.	F-prob.	SED
Sa	Kyela 2011	24	2.982	<0.001	0.030	9.477	<0.001	0.490	2.922	<0.001	0.077
	Kyela 2012-1	24	4.953	<0.001	0.025	4.172	<0.001	0.496	1.255	0.217	0.096
	Kyela 2012-2	24	3.058	<0.001	0.026	75.408	<0.001	0.304	2.017	0.009	2.430

Sh	Mbita 2010	24 (23)^c^	1.881	0.019	0.048	11.115	<0.001	1.060	1.946	0.012	5.008
	Mbita 2011	24 (23)^c^	1.256	0.221	0.043	55.181	<0.001	0.627	5.548	<0.001	11.100

a Sa = *S. asiatica*; Sh = *S. hermonthica*.

b Based on a generalized linear model with Poisson distribution.

c Grain DW of Mbita trials have 23 degrees of freedom, as Supa India did not reach flowering due to photoperiodicity.

In all the field trials, mean maximum above-ground *Striga* numbers (*NSmax*) per cultivar correlated positively and highly significantly (*P* < 0.001) with the mean *Striga* dry weights at harvest per cultivar (Spearman correlation coefficients for *S. asiatica* were *r*_2011_ = 0.77, *r*_2012-1_ = 0.73, and *r*_2012-2_ = 0.76; for *S. hermonthica* correlation coefficients were *r*_2010_ = 0.88 and *r*_2011_ = 0.89).

**Fig. 1:**
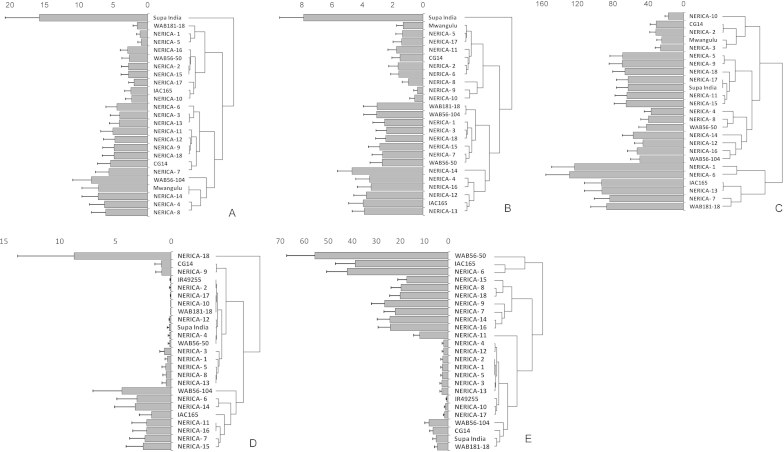
Maximum number of emerged *Striga* plants m^−2^ per cultivar for Kyela 2011 (A), 2012-1 (B) and 2012-2 (C) – *S. asiatica* – and for Mbita 2010 (D) and 2011 (E) – *S. hermonthica*. Left side: means and standard errors of means; right side: cluster analyses.

Based on maximum above-ground *S. asiatica* numbers (*NSmax*) observed in the field, and using the hierarchical cluster analysis and evaluation measures outlined above, rice cultivars were clustered into three groups, representing different resistance levels in each field trial in Kyela (Fig. 1A–C). In the 2011 field trial, the cluster with the most resistant cultivars, comprised NERICA-1, -5, -2, -10, -15, -16 and -17, WAB181-18, WAB56-50 and IAC165 (Fig. 1A). For the Kyela 2012 -1 field trial (the same field as the 2011 trial), the most resistant cultivars were NERICA-8, -9, -10, -2, -5, -6, -11 and -17 and CG14 and Mwangulu (Fig. 1B). The cultivars at the second field site (Kyela 2012-2) had much higher *Striga* infestation levels compared to those at the Kyela 2012-1 field site (Fig. 1B and C). The most resistant cultivars in the Kyela 2012-2 trial were NERICA-2, -3 -10, -4, -8, -12, -14 and -16 and CG14, Mwangulu, WAB56-50 and WAB56-104 (Fig. 1C). Table 3, summarizing the groupings based on *NSmax* using cluster analysis, shows that the cultivars expressing consistently high levels of field resistance against *S. asiatica* across the two seasons and sites were NERICA-2 -10, -5 and -17.

**Table 3:** Summary of hierarchical cluster analyses^a^ based on maximum above-ground *Striga* numbers (*NSmax*), a measure for resistance in the field, observed in the *S. asiatica*-infested field in Kyela, Tanzania (2011, 2012-1 and 2012-2) and the *S. hermonthica*-infested field in Mbita, Kenya (2010 and 2011); cluster 1 groups together the most resistant cultivars of a particular screening trial, cluster 2 groups cultivars of intermediate resistance/susceptibility and cluster 3 represents cultivars with susceptibility to a particular *Striga* species. For each cluster, the mean *NSmax* (in number of plants m^−2^) is shown. Underlined are names of cultivars showing consistent good resistance against *Striga* sp.

Cluster	*S. asiatica*			*S. hermonthica*	
	2011	2012-1	2012–2	2010	2011
Resistant					
1	N1^b^, N5, N2, N10, N15, N16, N17, WAB181-18, WAB56-50, IAC165	N8, N9, N10, N2, N5, N6, N11, N17, CG14, Mwangulu	N2, N3, N10, N4, N8, N12, N14, N16, N5, N9, N11, N15, N17, N18, CG14, Mwangulu, WAB56-50, WAB56-104, Supa India	N2, N4, N10,N12, N17, N1, N3, N5, N8, N13, N9, WAB181-18, WAB56-50, Supa India, IR49255-B-B-5-2, CG14	N1, N2, N3, N4, N5, N10, N12, N13, N17, N11, IR49255-B-B-5-2, WAB181-18, WAB56-104, CG14, Supa India
Mean	2.1	1.3	48.0	0.3	3.6

Intermediate					
2	N3, N6, N7, N9, N11, N12, N13, N18, N8, N4, N14, CG14, Mwangulu, WAB56-104	N1, N3, N7, N15, N18, N4, N12, N13, N14, N16, WAB56-50, WAB56-104, WAB181-18, IAC165	N7, N13, IAC165, WAB181-18	N6, N7, N11, N14, N15, N16, WAB56-104, IAC165	N7, N8, N9, N14, N15, N16, N18
Mean	5.6	3.2	88.9	2.7	22.0

Susceptible					
3	Supa India	Supa India	N1, N6	N18	N6, WAB56-50, IAC165
Mean	15.7	7.9	126.1	8.7	45.4

a The Connectivity (Handl et al., 2005), Dunn Index (Dunn, 1974), and Silhouette Width (Rousseeuw, 1987) measures are used to optimize the number of clusters (see Section 2.6).

b NERICA cultivars are abbreviated by ‘N’ following the specific number.

Based on maximum above-ground numbers of *S. hermonthica* (*NSmax*), and using the hierarchical cluster analysis and evaluation measures outlined above, rice cultivars were clustered again into three groups in both field trials carried out in Mbita (Fig. 1D and E). In 2010, the most resistant cultivars comprised NERICA-2, -4, -10, -12, -17, -1, -3, -5, -8 and -13 and WAB181-18, WAB56-50, Supa India and IR49255-B-B-5-2 (Fig. 1D). In the second season (2011) the *Striga* infection levels were much higher than in the first season. Here the most resistant cultivars were NERICA-1, -2, -3, -4, -5, -10, -12, -13, -17 and -11, and IR49255-B-B-5-2, WAB181-18, WAB56-50, CG14 and Supa India (Fig. 1E). Following the cluster analysis, the cultivars expressing consistently high levels of field resistance against *S. hermonthica* were NERICA-1, -2, -3, -4, -5, -10, -12, -13 and -17, and IR49255-B-B-5-2, CG14 and Supa India (Table 3).

**Fig. 2:**
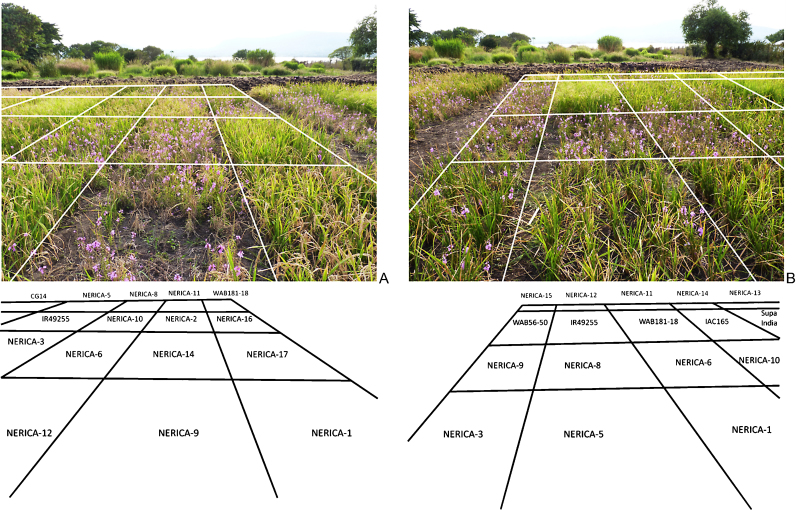
Contrasting *Striga* infection levels in the *S. hermonthica* screening trial at Mbita, Kenya (July 2011) replicate 6 (A) and replicate 3 (B); Sub-plots, representing cultivars are delimited by white lines.

The variation in resistance to *S. hermonthica* among cultivars is illustrated in Fig. 2, which shows plots within two adjacent replicates of the Mbita 2011 trial. NERICA-1, -10 and -17 have little emerged *Striga* whereas NERICA-14 and -9 are very susceptible and highly infected by *S. hermonthica*. Most of the NERICA-9 rice plants in this replicate have died because of the high infection levels (Fig. 2A). The number of *Striga* plants parasitizing susceptible cultivars WAB56-50 and NERICA-8 and -6 contrasts with the good resistance of NERICA-1, -3 and -5 and the cultivar IR49255-B-B-5-2 (Fig. 2B).

### 3.2 Rice grain yields under *S. hermonthica* and *S. asiatica* infestation

**Fig. 3:**
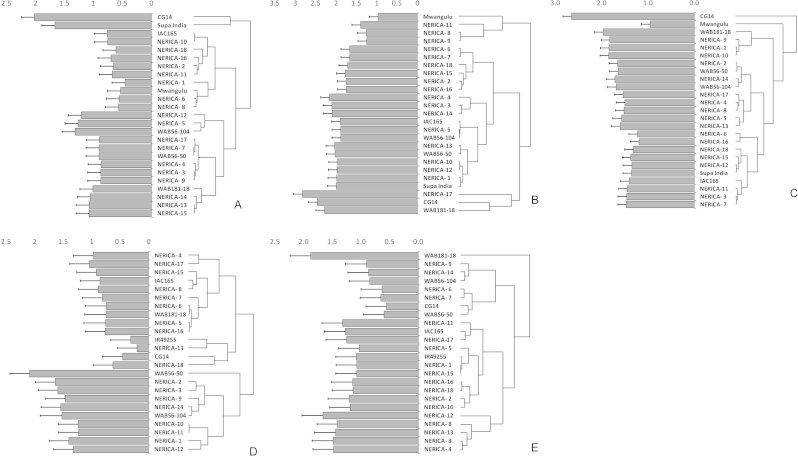
Rice grain dry weights (t ha^−1^) per cultivar for Kyela 2011 (A), 2012-1 (B) and 2012-2 (C) – *S. asiatica* – and for Mbita 2010 (D) and 2011 (E) – *S. hermonthica*. Left side: means and standard errors of means; right side: cluster analyses.

For both locations (Kyela and Mbita) the Trial × Cultivar interaction effects on rice grain dry weights were significant (Kyela: *P* = 0.0013; Mbita: *P* = 0.0207), and therefore data were analyzed separately for each field trial. With a few exceptions the extrapolated rice grain yields obtained in both the *S. asiatica* and the *S. hermonthica*-infested fields did not exceed 2 t ha^−1^ (Fig. 3). In the *S. asiatica* infested fields, average yield per cultivar ranged from 0.4 to 2.0 t ha^−1^ (average: 0.9 t ha^−1^) in 2011, and from 0.9 to 2.8 t ha^−1^ (average: 1.9 t ha^−1^) and 1.0 to 2.6 t ha^−1^ (average: 1.6 t ha^−1^) in 2012 (fields 1 and 2 respectively). Based on rice grain weights obtained under *S. asiatica* infested conditions, and using the hierarchical cluster analysis and evaluation measures outlined above, cultivars were clustered in three groups (Fig. 3A–C). In 2011, the highest yielding cultivars were CG14 and Supa India (Fig. 3A). The second-highest yielding group consisted of NERICA-5, -12, -3, -4, -7, -9, -13, -14, -15 and -17 and WAB56-104, WAB56-50 and WAB181-18. Cultivars in the remaining clusters yielded well below 1 t ha^−1^. The cluster with the highest yielding cultivars in the 2012-1 trial contained NERICA-17, CG14 and WAB181-18 (Fig. 3B). The third cluster included NERICA-8, -9 and -11 and Mwangulu and the second cluster included all other cultivars. In the second *S. asiatica* screening trial conducted in 2012, the highest yielding cultivar was again CG14, with well over 2 t ha^−1^ (Fig. 3C). The lowest yielding cultivar, in cluster 3, was Mwangulu. The second cluster included all other cultivars, with an average yield of 1.5 t ha^−1^. The only cultivar expressing consistent high levels of grain yield under *S. asiatica* infested conditions was CG14 (Table 4).

**Table 4:** Summary of hierarchical cluster analyses^a^ based on rice grain yields (at 14% grain moisture content), measured in the *S. asiatica*-infested field in Kyela, Tanzania (2011, 2012-1 and 2012-2) and the *S. hermonthica*-infested field in Mbita, Kenya (2010 and 2011); cluster 1 groups together the highest yielding cultivars of a particular screening trial, cluster 2 groups cultivars with intermediate high yields, cluster 3 represents cultivars of intermediate low yields and clusters 4 and 5 groups low yielding cultivars. Underlined are names of cultivars showing consistent good yields under *Striga* sp. infestation. For each cluster, the mean extrapolated rice grain yield (in t ha^−1^) is shown.

Cluster	*S. asiatica*			*S. hermonthica*	
	2011	2012-1	2012-2	2010	2011
High yielding					
1	Supa India, CG14	N17^b^, CG14, WAB181-18	CG14	WAB56-50	WAB181-18
Mean	1.8	2.5	2.7	2.1	1.9

Intermediate-high yielding					
2	N5, N12, N3, N4, N7, N9, N13, N14, N15, N17, WAB56-104, WAB56-50, WAB181-18	N3, N4, N14, N1, N5, N10, N12, N13, N2, N6, N7, N15, N16, N18, WAB56-50, WAB56-104, IAC165, Supa India	N1, N9, N10, WAB181-18, N2, N4, N5, N8, N13, N14, N17, N3, N6, N7, N11, N12, N15, N16, N18, WAB56-50, WAB56-104, Supa India, IAC165	N1, N2, N3, N9, N10, N11, N12, N14, WAB56-104	N3, N4, N8, N12, N13
Mean	1.0	1.9	1.5	1.4	1.5

Intermediate-low yielding					
3	N2, N10, N11, N16, N18, IAC165, N1, N6, N8, Mwangulu	N8, N9, N11, Mwangulu	Mwangulu	N4, N5, N6, N7, N8, N15, N16- N17, N13, N18, WAB181-18, IAC165, CG14, IR49255-B-B-5-2	N1, N2, N5, N10, N11, N15, N16, N17, N18, IAC165, IR49255-B-B-5-2
Mean	0.6	1.2	1.0	0.7	1.2

Low yielding					
4					N9, N14, WAB56-104
Mean					0.9
5					N6, N7, WAB56-50, CG14
Mean					0.6

a The Connectivity (Handl et al., 2005), Dunn Index (Dunn, 1974), and Silhouette Width (Rousseeuw, 1987) measures are used to optimize the number of clusters (see Section 2.6).

b NERICA cultivars are abbreviated by ‘N’ following the specific number.

In the *S. hermonthica*-infested fields, in Mbita, based on grain yields and using the hierarchical cluster analysis and evaluation measures outlined above, cultivars were clustered into three (2010) and five (2011) groups (Fig. 3D and E). Averaged across the cultivars, the yield in 2011 (1.1 t ha^−1^) was similar to that in 2010 (1.0 t ha^−1^), but the variability in average yield among cultivars was higher in 2010 (ranging from 0.2 to 2.1 t ha^−1^) than in 2011 (ranging from 0.5 to 1.9 t ha^−1^). The cluster with best yielding cultivars in 2010 contained only WAB56-50. This was followed by a cluster of NERICA-1 to -3, NERICA-9 to -12 and NERICA-14 and WAB56-104. In 2011, the highest yielding cultivar was WAB181-18. The cluster with second-highest yielding cultivars included NERICA-3, -4, -8, -12 and -13. It was closely followed by a third cluster containing NERICA-1, -2, -5, -10, -11, and NERICA-15 to -18, IAC165 and IR49255-B-B-5-2. The cultivars expressing consistent high levels of grain yield under *S. hermonthica* infested conditions were NERICA-3 and -12, (Table 4).

### 3.3 Is there a relationship between rice grain yield and resistance to *Striga*?

**Fig. 4:**
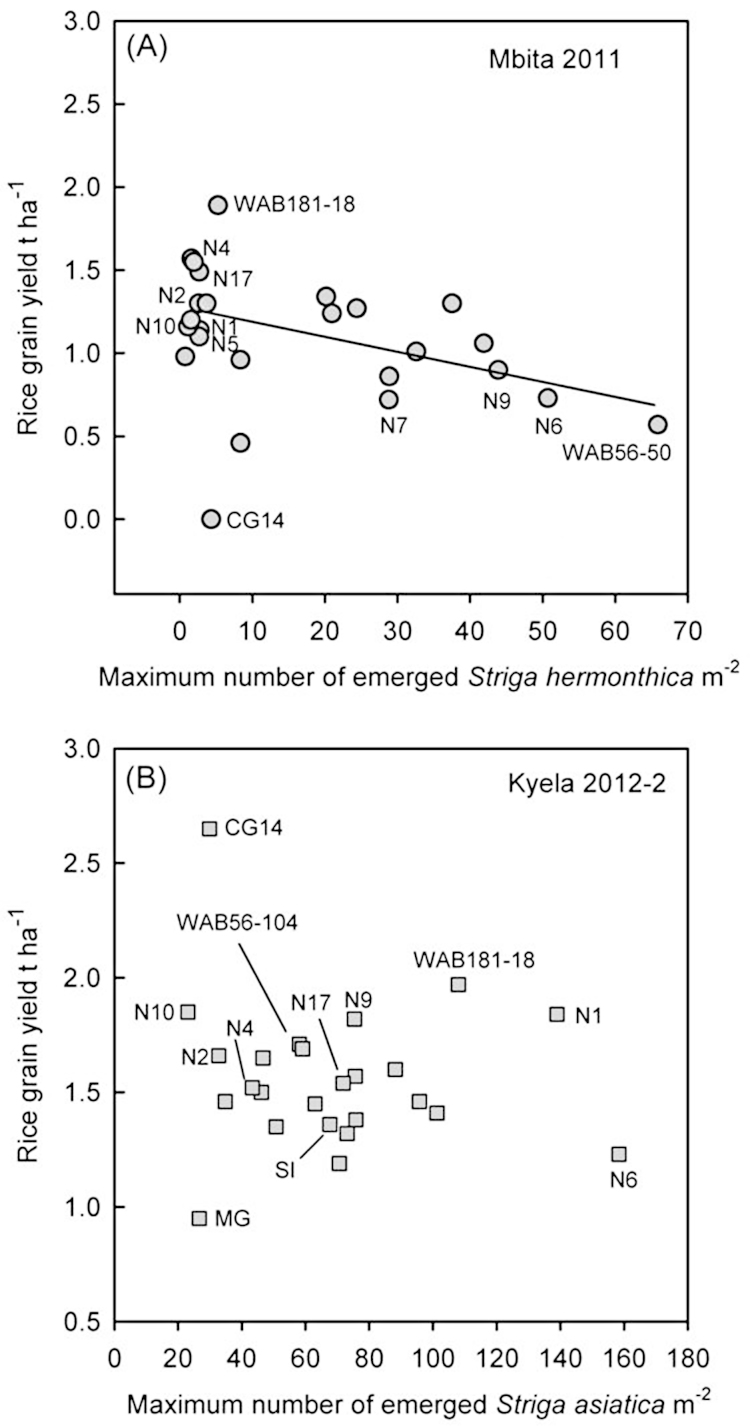
The relationship between resistance of the cultivars (maximum number of emerged *Striga* m^−2^) and rice grain yield (t ha^−1^). (A) Mbita field trial 2011 (*R*^2^ = −0.28); (B) Kyela field trial 2012-2. NERICA cultivars are abbreviated by ‘N’ following the specific number, Mwangulu is abbreviated as ‘MG’ and Supa India as ‘SI’.

In the 2011 field trial at Mbita (when *S. hermonthica* infection levels were high) there was a moderate but significant correlation between the resistance ranking of the cultivars, based on *NSmax*, and grain yield (*r* = −0.45; *P* = 0.027) with the more resistant cultivars showing the greatest grain yields (Fig. 4A). In 2010, when infection levels were low, no such relationship was seen. In the field trials at Kyela there was no consistent pattern between the level of resistance to *S. asiatica* and grain yield as illustrated by data from Kyela 2012-2, the trial with the highest *S. asiatica* infection levels (Fig. 4B). In some cases cultivars with good resistance had some of the highest yields whereas others had yields that were similar to more susceptible cultivars (Fig. 1, Fig. 3, Fig. 4). Interestingly, in Kyela, CG14 showed very good resistance to *S. asiatica* under both low (2011 and 2012-1) and high (2012-2) infestation levels and achieved the highest yield (greater than 2 t ha^−1^) each year. However, although CG14 also showed good resistance in both trials at Mbita, it yielded poorly in that site (Fig. 1, Fig. 3, Fig. 4).

### 3.4 How resistant are the NERICA cultivars and their parental genotypes to the *S. hermonthica* (Sh-Mbita) and *S. asiatica* (Sa-Kyela) ecotypes under controlled environment conditions?

**Fig. 5:**
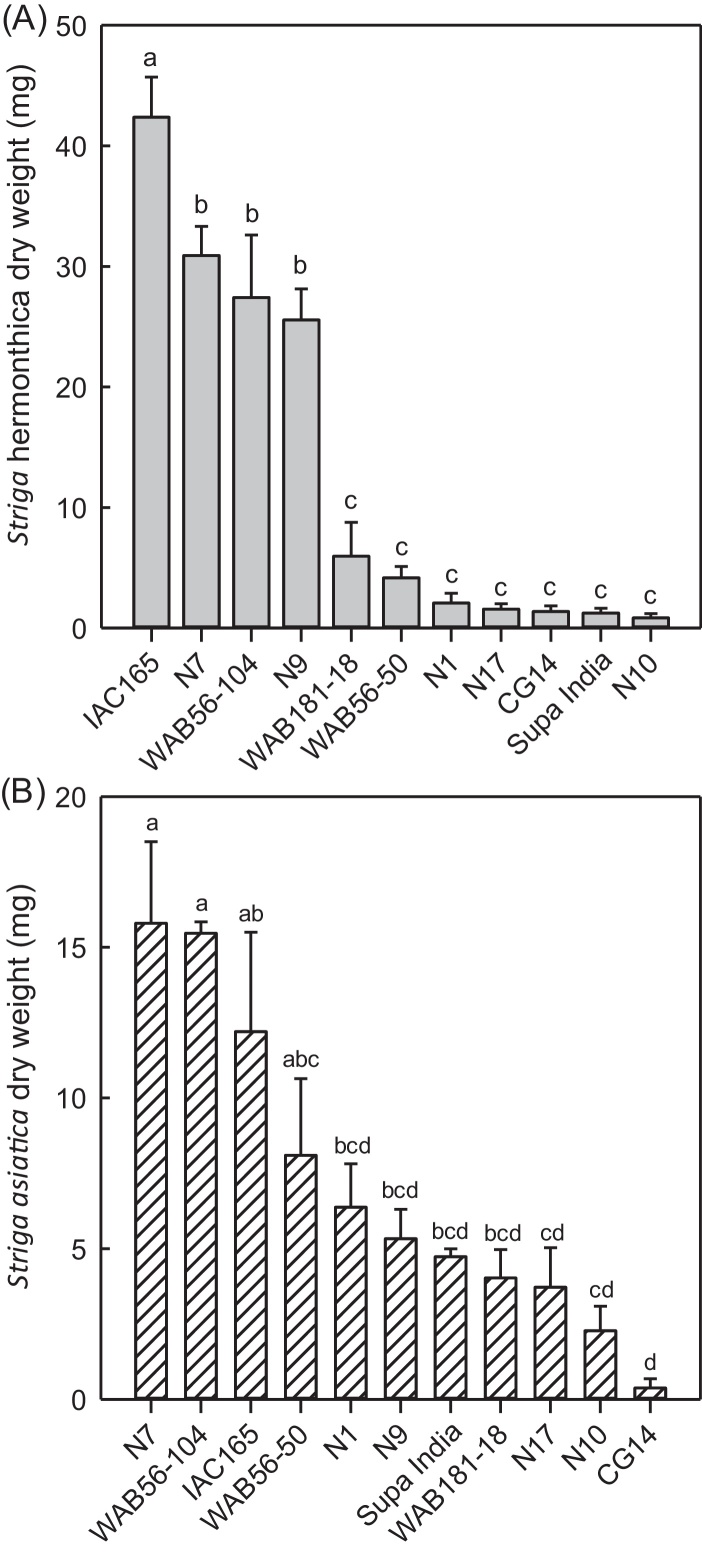
Post attachment resistance of selected NERICA rice cultivars (N1, N7, N9, N10 and N17) and their parents to (A) *Striga hermonthica* (Sh-Mbita) and (B) *S. asiatica* (Sa-Kyela) ecotypes collected from the field sites at Mbita Point, Kenya and Kyela, Tanzania respectively. *Striga* dry weight was assessed at 21 days after infection. Data are means of four replicates ± SE. Means with the same letter do not differ significantly from each other (Tukey multiple comparison test, *P* > 0.05).

A significant cultivar effect on *S. hermonthica* (Sh-Mbita) infection levels (*F* = 57.1, df = 10, *P* = 0.001) was observed under controlled environment conditions (in rhizotrons) with WAB181-18, WAB56-50, CG14, Supa India and NERICA-1, -10 and -17 exhibiting good resistance (Fig. 5A). The most resistant cultivars had few successful attachments resulting in low parasite biomass on the roots. IAC165, WAB56-104 and NERICA-7 and -9 were very susceptible with a large number of attachments and high parasite biomass. A significant cultivar effect on *S. asiatica* (Sa-Kyela) infection levels (*F* = 11.0, df = 10, *P* = 0.001) was also observed. The most resistant cultivars were CG14, NERICA-10 and -17 supporting few attachments and low *Striga* biomass (Fig. 5B) whilst the most susceptible were NERICA-7, WAB56-104, WAB56-50 and IAC165, supporting the largest number and biomass of parasites on their roots.

**Fig. 6:**
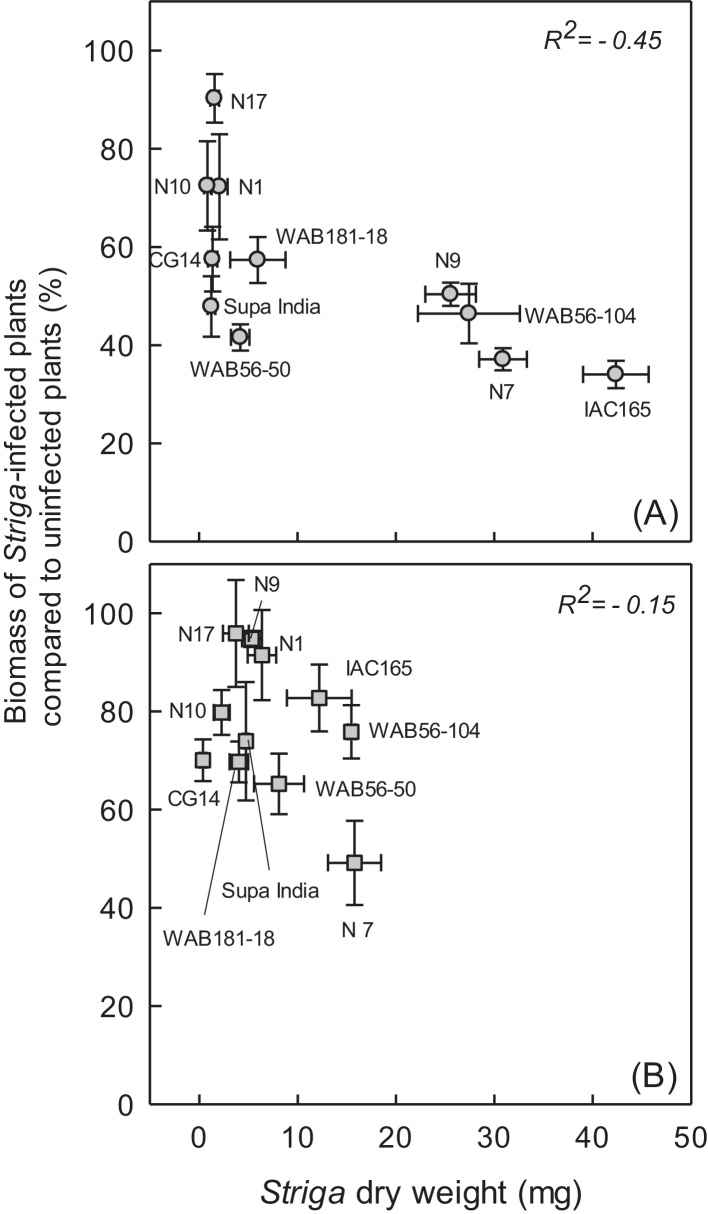
Relationship between the biomass of *Striga*-infected plants compared to uninfected plants (%) and the dry weight of (A) *Striga hermonthica* (Sh-Mbita) and (B) *S. asiatica* (Sa-Kyela), attached to the roots of NERICA rice cultivars (N1, N7, N9, N10 and N17), parental lines and checks, 21 days after infection. Data are presented as means ± SE of four replicates.

With both *S. hermonthica* and *S. asiatica*, there was a negative relation between the parasitic biomass on the roots and the percentage biomass of infected host-plants compared to uninfected control plants (Fig. 6). The most resistant cultivars (NERICA-17, -10 and -1) only showed a small (10–25%) reduction in biomass compared with the uninfected controls. This contrasted with the most susceptible cultivars, NERICA-9, -7, WAB56-104 and IAC165, which all lost 50–65% of their biomass compared to their respective control plants, when infected with either *S. hermonthica* or *S. asiatica* (Fig. 6). There was also a difference in growth performance (tolerance) between cultivars subjected to the same amount of *Striga* infection. For example, NERICA-17 showed 10% reduction in biomass when infected by *S. hermonthica* or *S. asiatica*, while Supa India showed 40 and 50% at similar infection levels of *S. hermonthica* and *S. asiatica* respectively.

## 4 Discussion

The interspecific NERICA cultivars have been widely adopted by farmers in rain-fed upland rice growing areas in sub-Saharan Africa (Wopereis et al., 2008). Recently however, mixed levels of the resistance of different NERICA cultivars have been reported (J. Rodenburg, personal observation). In 2011 Jamil et al., and Cissoko et al., analyzed pre-and post-attachment resistance levels of the 18 upland NERICA cultivars and their parental genotypes to different *Striga* species and ecotypes under controlled environment growth conditions. They found that some NERICA cultivars showed good pre- and/or post-attachment resistance against different *Striga* species and ecotypes whereas others only showed resistance against a specific species or ecotype and some were susceptible (to varying degrees) to all *Striga* ecotypes. However, the impact of the environment on the expression of these host resistance mechanisms (i.e. pre- and post-attachment) and the adaptability and yield of different cultivars under *Striga*-infested field conditions is largely unknown. The only previously published study, conducted by Atera et al. (2012) with a small selection of NERICA cultivars, showed that NERICA-1 and NERICA-10 yielded anywhere between 1.7 and 2.5 t ha^−1^ under *S. hermonthica*-infested field conditions in Kenya. Atera et al. (2012), did however no provide any information on *Striga* infection levels hence no inference could be drawn on the *Striga* resistance or tolerance levels of the rice cultivars under review. Information on resistance and yield levels under field conditions is of paramount importance to farmers when selecting cultivars for different agro-ecological zones.

### 4.1 How resistant are the NERICA rice cultivars to *Striga* spp. in the field? Is there a correlation between resistance rankings obtained under controlled environment conditions and in the field?

Among the set of 25 rice cultivars screened under field conditions in Tanzania and Kenya, significant differences were found in their levels of resistance against *S. asiatica* and *S. hermonthica* as summarized in Table 5. Nine of the 18 NERICA cultivars (NERICA 1-5, -10, -12, -13 and -17), one of the three *O. sativa* parents (WAB181-18) and the *O. glaberrima* parent (CG14) showed good or excellent resistance to the *S. hermonthica* ecotype from Mbita in the field. The same nine NERICA cultivars were also ranked as the most resistant (post-attachment resistance) to the *S. hermonthica* ecotype from Kibos, western Kenya (Sh-Kibos) in a previous rhizotron (controlled environment) study by Cissoko et al. (2011). For the selected cultivars we tested in the current study in a rhizotron, with the same *S. hermonthica* ecotype as the one present in the field (Sh-Mbita), resistance found in the field was confirmed. Many of these same cultivars (i.e. NERICA-1, -3, -4, -12 and -17, CG14 and WAB181-18) also had good pre-attachment resistance to an ecotype of *S. hermonthica* from Medani (Sudan) (see: Jamil et al., 2011b).

**Table 5:** Summary of the resistance levels of rice cultivars to *S. hermonthica* and *S. asiatica* ecotypes in field and controlled environments (pre and post-attachment resistance). Cultivars are ranked Resistant (R), Susceptible (S) or Intermediate (I); based on *NSmax* (field) or the average number of attached *Striga* plants (controlled environment).

Cultivar/*Striga* ecotype	Field		Controlled environment				
			Post-attachment^b^				Pre-attachment resistance^b^
	Sh-Mb^a^	Sa-Ky	Sh-Mb	Sa-Ky	Sh-Ki	Sa-US	Sh-Me
NERICA-1	R	I/S	R	I	R	R	R
NERICA-2	R	R	–	–	R	R	I
NERICA-3	R	R	–	–	R	R	R
NERICA-4	R	I/S	–	–	R	R	R
NERICA-5	R	R	–	–	R	R	I
NERICA-6	S	I/S	–	–	S	S	I
NERICA-7	S	S	S	S	S	S	S
NERICA-8	I	I/R	–	–	S	S	S
NERICA-9	I/S	I/S	S	I	S	I	I
NERICA-10	R	R	R	R	R	R	I
NERICA-11	I/S	I/S	–	–	S	S	S
NERICA-12	R	I	–	–	R	R	R
NERICA-13	R	S	–	–	I	R	I
NERICA-14	S	S	–	–	S	S	S
NERICA-15	S	I/S	–	–	S	S	I
NERICA-16	S	I/S	–	–	S	S	R
NERICA-17	R	R	R	R	R	R	R
NERICA-18	S	I/S	–	–	S	S	I
CG14	R	R	R	R	R	R	R
WAB56-104	I/S	I	S	S	I	I	R
WAB56-50	I/S	I	R	I	I	R	S
WAB181-18	R	I	R	R	I	R	R
IAC165	S	S	S	S	S	S	–
Supa India	R	S	R	I	–	–	–

a *Striga* ecotypes: Sh-Mb = *S. hermonthica* from Mbita (Kenya); Sh-Ki = *S. hermonthica* from Kibos (Kenya); Sh-Me = *S. hermonthica* from Medani (Sudan); Sa-Ky = *S. asiatica* from Kyela (Tanzania); Sa-US = *S. asiatica* from USA. IR49255-B-B-2 and Mwangulu are not shown as they were only tested in one field site and not in controlled environments.

b Information on post-attachment resistance is derived from Cissoko et al. (2011) and on pre-attachment resistance from Jamil et al. (2011b).

This suggests that these NERICA cultivars have broad-spectrum resistance to at least several *S. hermonthica* ecotypes. IR49255-B-B-5-2 which was used as resistant ‘check cultivar’ in this study also exhibited a good level of resistance to Sh-Mbita confirming previous field and pot studies where this cultivar was highly resistant to other *S. hermonthica* ecotypes (Harahap et al., 1993, Johnson et al., 1997).

The classification of *S. asiatica* resistance in the field seems to be highly dependent on the *Striga* infection levels, as NERICA-5 and -17, for instance, showed relatively high field resistance against *S. asiatica* under moderate to low infection levels (2011 and 2012-1 trials) but were more susceptible under the high infection levels of the 2012-2 field. Only three NERICA cultivars (NERICA-2, -3 and -10) and the *O. glaberrima* parent CG14 showed very good field resistance to Sa-Kyela under high infestation levels (in the 2012-2 trial) although several others e.g. NERICA-4, -8, -12, -14 and -16, as well as NERICA-5, -9, -11, -15 -17 and -18 showed intermediate resistance. Of the above mentioned cultivars NERICA-10 and -17 and CG14 were assessed against Sa-Kyela, for post-attachment resistance, under controlled environment conditions where they also exhibited good resistance (Table 5). Field resistance of NERICA-2, -3, -4, -10, -12 and -17 and CG14 confirmed the post-attachment resistance ranks based on a previous rhizotron study by Cissoko et al. (2011), with a *S. asiatica* ecotype from the USA (Table 5).

Supa India was very susceptible to Sa-Kyela but resistant to Sh-Mbita. This cultivar has been grown for many years by the farmers at Kyela and it is likely that the virulence levels of the local parasite population against this cultivar have increased in time. Supa India had not been grown at Mbita prior to this study and showed good resistance to *S. hermonthica.* Based on insights presented by Huang et al. (2012), this would suggest that the *S. hermonthica* population in this field did not have the virulence loci to overcome resistance in this cultivar. It is also interesting to note that many of the NERICA cultivars exhibited different resistance levels (in both field and controlled environment studies) when infected with Sh-Mbita compared to Sa-Kyela. For example NERICA-14 and -16 were very susceptible to Sh-Mbita but showed intermediate resistance under high infestation levels of Sa-Kyela and NERICA-1 and -13 showed good resistance to Sh-Mbita but were susceptible to Sa-Kyela. These differences in host-parasite specificity again suggest that the ecotypes of these two species of *Striga* have very different suites of virulence loci.

Although the correspondence between the resistance levels of the cultivars when screened in the field and under controlled environment conditions was remarkably good, they were not always exact. For example, WAB56-50 proved susceptible against *S. hermonthica* in the field, but resistant against the same ecotype in the rhizotron (i.e. in the post-attachment stage). NERICA-8, -14 and -16 showed intermediate resistance against *S. asiatica* in the field, but proved susceptible to Sa-USA in the rhizotron study by Cissoko et al. (2011). WAB56-104 was susceptible against *S. asiatica* in the rhizotron but had intermediate resistance in the field, while the reverse situation was observed with WAB181-18. Such differences confirm earlier findings that resistance observed under controlled environments do not always express in exactly the same way in the field (e.g. Omanya et al., 2004). There are a number of reasons for this including, differences in *Striga* infestation level and non-homogenous distribution of seeds in the soil (Haussmann et al., 2000), variability in soil fertility (particularly P and N), which may affect the production of strigolactones by the host roots and hence the germination of *Striga* seeds (Yoneyama et al., 2007, Jamil et al., 2011a, Umehara, 2011) and the soil moisture, flora and fauna of infested fields. All these factors create a different screening environment compared to the fully controlled situations in the laboratory (e.g. Haussmann et al., 2000). In addition, the characteristics of the host root system play a role in the responses of cultivars to *Striga* infection in the field. Cultivars with simpler, less branched roots can avoid or escape *Striga* parasitism in the field (Arnaud et al., 1999, Delft et al., 1996) and thus have fewer attached parasites. In the rhizotron study, *Striga* seeds were aligned along the host roots even if apparent differences are observed in root morphology or architecture of rice cultivars tested.

### 4.2 Do rice cultivars that exhibit good resistance responses in the field also produce good yields under *Striga* infested conditions?

Rice cultivars with good resistance are suitable for rice production in sub-Saharan Africa where *S. hermonthica* and *S. asiatica* are prevalent, provided that they are adapted to the prevailing growing environments (Rodenburg et al., 2010). Environmental adaptation is reflected in growth and reproduction parameters, such as biomass and yield. Rice grain weights (at 14% moisture content) showed a significant negative correlation with maximum above-ground *Striga* numbers (*NSmax*), as a measure for susceptibility (i.e. in general the most resistant cultivars produced the highest yields), only in the 2011 trial in Mbita, under high *S. hermonthica* pressure. This is in line with earlier field screening results with sorghum cultivars, where only under high *S. hermonthica* infestation the negative correlation between *Striga* numbers and yield under *Striga* infestation appears significant (Rodenburg et al., 2005). This result would imply that *Striga* resistance only provides a yield advantage under high infection levels.

Under *Striga*-infested conditions the best performing rice cultivars yielded an equivalent of 1.5–2.5 t ha^−1^ at Mbita (e.g. WAB56-50, NERICA-2 and -3 in 2010; WAB181-18, NERICA-3, -4 and -12 in 2011) and at Kyela (e.g. CG14 and Supa India in 2011; CG14, WAB181-18 and NERICA-17, -3, -4 and -14 in 2012-1; CG14, WAB181-18 and NERICA-1, -9 and -10 in 2012-2). These yields were similar to experimentally obtained upland rice yields of sub-optimally weeded plots (e.g. Ekeleme et al., 2009, Toure et al., 2011) or in sub-optimally fertilized plots (e.g. Saito et al., 2012) elsewhere in SSA. Yields of the best performing NERICA cultivars were mostly higher than the overall average estimated yield of upland rice (i.e. 1–1.25 t ha^−1^) obtained by farmers in Eastern Africa (e.g. Mghase et al., 2010, Sekiya et al., 2013) as well as the wider region (e.g. Seck et al., 2012, Diagne et al., 2013).

Cultivar performance in the two field trials is probably not only limited by *Striga* parasitism. The two field sites (particularly Kyela) are characterized by poor soil fertility, caused by continuous crop production without nutrient replenishment by appropriate fertilizer applications. Confirming our own soil fertility assessments, soils in Kyela are characterized by 0.16% N and around 5 ppm of available P (Mghase et al., 2010) while soils in Mbita have been reported to have 0.09–0.12% N and 6.3–13.3 ppm available P (Weisskopf et al., 2009). This relative poor soil fertility has certainly negatively affected crop performance, in particular in Kyela. The optimization of crop performance, including that of NERICA rice cultivars, in these nutrient-limited soils will require a good management and application of fertilizers (e.g. Saito and Futakuchi, 2009). Improving soil fertility will also improve performance of rice in *Striga*-infested fields as shown by Adagba et al*.* (2002) where an application of 90–120 kg N ha^−1^ helped to reduce the number of emerged *Striga* plants and boost rice yields.

Under *Striga*-infested field conditions, *Striga* resistance may have an important contribution to satisfactory yields, but final crop yields will depend on a suite of other genetic and non-genetic factors and interactions. The highly resistant rice cultivars IR49255-B-B-5-2 and CG14, for instance, showed lower grain yields at Mbita than some of the resistant NERICA cultivars (e.g. -3, -4, -12, and -13) despite similar infection levels. This may be caused by differences in *Striga* tolerance, a general lower level of genetically determined yield potential, or differences in environmental adaptation. The interspecific NERICA cultivars are known to combine relatively high yields with overall good adaptability to rain-fed upland environments (e.g. Saito et al., 2012). While for IR49255-B-B-5-2, only tested at one field site, causes for the poor yields cannot be conclusively established based on data presented here, the poor performance of CG14 under *S. hermonthica* infestation in Mbita must be a result to the lack of adaptability to the prevailing growing conditions at that site; CG14 was the highest yielding cultivar under *S. asiatica* infestation in Kyela and the rhizotron study with the two ecotypes of these *Striga* species did not reveal any differences in tolerance of CG14 to any of these species.

### 4.3 Can rice cultivars be differentiated based on variation in tolerance against *Striga* spp.?

The difference in yield between equally resistant or equally susceptible cultivars observed in the field may be due to inherent genetic differences in levels of *Striga* tolerance, the physiological capacity of the host plant to alleviate parasitism effects, as previously shown in sorghum (e.g. Rodenburg et al., 2005, Rodenburg et al., 2006, Rodenburg et al., 2008). Revealing such traits requires a combination of *Striga*-free and *Striga*-infested plots in the experimental design. While in our study, no *Striga*-free control plants were grown under the same field conditions, both uninfected and infected plants were grown in the rhizotrons, which allowed us to compare tolerance of different cultivars, provided that they had similar infection levels. At similar *S. hermonthica* infection levels, NERICA-17, was markedly less affected by parasitism than NERICA-10, which in turn performed better than CG14 and Supa India. The same was observed when these cultivars were infected by *S. asiatica*. At higher infection levels, WAB56-104 performed better than NERICA-7. Such variation in tolerance levels in rice cultivars, reported before by Cissoko et al. (2011), should be further explored and exploited for breeding purposes. If tolerance can be introgressed into adapted, high yielding (with desirable grain quality) *Striga*-resistant cultivars, this trait will provide an additional safety net for farmers coping with *Striga* infested soils (Rodenburg and Bastiaans, 2011).

## 5 Conclusion

This study showed that some NERICA cultivars displayed good levels of resistance and tolerance to the two most important *Striga* species occurring in rain-fed cereal cropping systems. A number of NERICA cultivars, notably NERICA-2, -10, -5 and -17 for *S. asiatica* and NERICA-1, -2, -3, -4, -5, -10, -12, -13 and -17 for *S. hermonthica*, possessed superior resistance in the field. In addition, NERICA-1, -17 and -10 have been identified, in vitro, as cultivars with potentially good levels of *S. hermonthica* tolerance. Potential tolerance to *S. asiatica* has been observed in NERICA-1, -17 and -9, at low infection levels, and with WAB56-104, at high infection levels. These cultivars suffered less *Striga*-inflicted total plant biomass reduction compared to some other cultivars when subjected to similar *Striga* infection (biomass) levels. Yields obtained under *Striga*-infested conditions in the field show a high variability among cultivars, years and *Striga* species. However, under high parasite pressure reasonable yields were obtained by a number of NERICA cultivars, i.e. NERICA-1, -9 and -10 (under *S. asiatica* infestation) and NERICA-3, -4, -8, -12 and -13 (under *S. hermonthica* infestation).

This study showed that the use of in vitro methods to identify resistance based on single mechanisms (i.e. either pre-attachment or post-attachment) are useful for the identification of superior breeding material in particular when such methods are used in succession to identify material with resistance based on multiple mechanisms (i.e. pre- and post-attachment). The resistant cultivars identified in this study, could be used in breeding programs aiming at the development and improvement of *Striga* resistance in adapted and high yielding rice cultivars. Cultivars that combine such broad-based resistance with the ability to maintain satisfactory yield levels in the field (i.e. NERICA-10 for *S. asiatica*-infested fields and NERICA-3, -4, -13 and -12 for *S. hermonthica* infested fields) are also suitable for inclusion in an integrated *Striga* control program.

These findings are highly relevant to rice breeders and molecular geneticist working on *Striga* defence mechanisms, as well as to resource-poor rice farmers typically working in the poorly fertile, drought-prone and *Striga* infested upland ecosystems commonly found in sub-Saharan Africa.
